# SM22α deficiency: promoting vascular fibrosis via SRF-SMAD3-mediated activation of *Col1a2* transcription following arterial injury

**DOI:** 10.21203/rs.3.rs-3941602/v1

**Published:** 2024-02-27

**Authors:** Jianbin Shen, Donghong Ju, Shichao Wu, Jiawei Zhao, Lucynda Pham, Alejandro Ponce, Maozhou Yang, Hui Joyce Li, Kezhong Zhang, Zhe Yang, Youming Xie, Li Li

**Affiliations:** Wayne State University School of Medicine; Wayne State University; Wayne State University; Wayne State University; Wayne State University; Wayne State University; Henry Ford Health System; University of Massachusetts Chan Medical School; Wayne State University; Wayne State University; Wayne State University; Wayne State University

**Keywords:** SM22/Tagln, vascular fibrosis, Cal1a2, transcription, SRF, Smad3

## Abstract

Vascular fibrosis, characterized by increased Type I collagen expression, significantly contributes to vascular remodeling. Our previous studies show that disrupting the expression of SM22α (aka SM22, Tagln) induces extensive vascular remodeling following arterial injury, involving oxidative stress, inflammation, and chondrogenesis within the vessel wall. This study aims to investigate the molecular mechanisms underlying the transcription of *Col1a2*, a key fibrotic extracellular matrix marker. We observed upregulation of COL1A2 in the arterial wall of *Sm22*^−/−^ mice following carotid injury. Bioinformatics and molecular analyses reveal that *Col1a2* transcription depends on a CArG box in the promoter, activated synergistically by SRF and SMAD3. Notably, we detected enhanced nuclear translocation of both SRF and SMAD3 in the smooth muscle cells of the injured carotid artery in *Sm22*^−/−^ mice. These findings demonstrate that SM22 deficiency regulates vascular fibrosis through the interaction of SRF and the SMAD3-mediated canonical TGF-β1 signal pathway, suggesting SM22α as a potential therapeutic target for preventing vascular fibrosis.

## Introduction

Vascular fibrosis, characterized by a thickened vessel wall due to excessive collagen deposition, is a significant factor in cardiovascular diseases such as hypertension, atherosclerosis, and aneurysm [1]. Vascular smooth muscle cell (VSMC) is a key player in the pathogenesis of vascular fibrosis. One distinct feature of VSMCs is the phenotypic plasticity in response to stress stimulation: VSMCs undergo transition from their physiological contractile form to various dedifferentiated states involved in proliferation, migration, oxidative stress, inflammation, and osteochondrogenesis [2–8].

VSMC phenotypic modulation is marked by the downregulation of contractile cytoskeletal proteins such as smooth muscle α actin, myosin heavy chain and SM22α (aka SM22, Transgelin) [3, 9, 10]. The downregulation of contractile proteins is an early event of pathogenesis of vascular disorders [11–13]. SM22 deficiency has been shown to actively drives phenotypic modulation of a variety of pathogenic processes involving proliferation, migration, oxidative stress, inflammation and chondrogenesis in response to arterial injury [14–19].

Since vascular fibrosis can be induced by inflammation and chondrogenesis [5, 20, 21], we examined the expression of a type I collagen Col1a2 (a brotic marker) in injury-induced vessel wall of *Sm22*^−/−^ mice. Here we report the high expression of *Col1a2* in arteries of *Sm22*^−/−^ mice in response to vascular injury. Mechanistically, we find that serum response factor (SRF) [22, 23] and its interacting partner SMAD3 [24] bind to the *Col1a2* locus to synergistically activates the transcription of *Col1a2* in the vessel wall. This study sheds light on the molecular mechanisms underlying vascular fibrosis induction.

## Materials and Methods

### Artery injury by carotid denudation.

The mouse carotid denudation protocol was approved by the Institutional Animal Care and Use Committee (IACUC) at Wayne State University. The animal procedures conform with the NIH guidelines (Guide for the care and use of laboratory animals). Generation and characterization of *Sm22*^−/−^ mice at mixed C57BL/6x SV129 genetic background as described previously [25]: this mouse strain (named as *Tagln*^*tm1LiW*^/Mmnc or SM22 knockout) was deposited in the Mutant Mouse Resource and Research Centers (MMRRC, ID#67375, SM22 Knockout) supported by NIH (Stock Number: 067375-UNC). The mouse carotid denudation injury model was described before [15, 16]. Briefly, carotid denudation was carried out on male *Sm22*^−/−^ mice and their wild type littermates of 18–20 weeks of age, as described [26]. The mice were sacrificed two weeks after surgery. The 3mm carotid segments covering the part from 2 mm to 5 mm proximal to the carotid bifurcation were harvested and embedded in OCT medium (Tissue-Tek). Around 100 frozen slides were made for each mouse with triplicate sections on each slide at 8 μm thickness. The carotids of ve *Sm22*^−/−^ mice and their *Sm22*^+/+^ littermates were stored separately in RNAlater reagent (Ambion) at 4°C for no more than 1 week before RNA extraction for qRT-PCR.

### Immunohistochemistry (IHC) analysis.

Six slides, in the order of one every 15 consecutive slides, from each mouse were subject to H&E staining to screen sections with most prominent inflammatory responses. Immunohistochemistry was performed on the properly selected consecutive frozen slides using VECTASTAIN Elite ABC Kit (Vectorlabs). Briefly, air-dried slides were fixed in methanol containing 0.3% H_2_O_2_ for 10 minutes and serum blocked for 20 minutes. The following incubation steps of primary antibody, secondary antibody, ABC reagent and DAB substrate were performed according to the manufacturer’s protocol. The slides were counterstained with hematoxylin. The primary antibodies (1:50 dilution) against procollagen COL1A2 (Santa Cruz, sc-8787), SRF (Santa Cruz, sc-335) and SMAD3 (Abcam, ab28379) were used. The secondary antibodies without the primary antibodies were used as the negative control.

### Morphometric analysis.

For each antibody, both 100X and 400X images were taken using a Leica DM4000B microscope. Images of adventitia and media were separated using Photoshope 7.0 software. Semi-quantitative analyses of positive signals in adventitia and media were performed on 100X images using color segmentation and integrative optical density function in the Image-Pro software.

### Analysis of the *Col1a2* promoter.

The transcriptional factor binding sites of the *Col1a2* promoter of Homo sapiens, Mus musculus, Norway rat, Canis lupus and Equus caballus were analyzed using bioinformatic tools from the Genomatix (http://www.genomatix.de/) and UCSC genome browser (https://genome.ucsc.edu/, 2019 year). The sequence of CArG box (the SRF binding site) in the promotor of *Col1a2* in Homo sapiens, Mus musculus, Norway rat, Canis lupus and Equus caballus were compared using VISTA (VISTA tools (lbl.gov)) and UCSC genome browser (https://genome.ucsc.edu/, 2019 year).

The ChIP-seq data analyses from GTRD-V20.06 database (GTRD (biouml.org)) and UCSC genome browser (https://genome.ucsc.edu/, 2019 year) were performed to confirm the binding of SRF and SMAD3 in the promotor region of *Col1a2* in Homo sapiens, Mus musculus, Norway rat, Canis lupus and Equus caballus. The sequences of these promoters were marked with the number started from the transcription starting site.

### Real-time RT-PCR (rtRT-PCR).

Total RNA from carotids was extracted and purified using RNeasy Fibrous Tissue Kit (Qiagen), and total RNA from tissue culture cells was extracted and purified using RNeasy Kit (Qiagen). The cDNA was synthesized using the Superscript II reverse transcriptase (Invitrogen). Real-time PCR was performed using SYBR Green on a StepOnePlus system (Applied Biosystems). GAPDH and snRNA U6 were used as internal controls in ΔΔCt method. All PCR primers were designed to cover at least 2 exons.

### Plasmid Constructions and Mutagenesis

The *Col2a1* reporter containing the 1342 bp of the mouse *Col2a1* gene was PCR cloned into the NheI/HindIII sites of pGL3-basic luciferase vector (Promega). The CC in the putative CArG box (CCAAACTTGG) was mutated to AT by PCR mutagenesis using the QuikChange^™^ site-directed mutagenesis kit (Stratagene, La Jolla, CA). Luciferase reporter plasmids were prepared for transfection using the plasmid maxi kit (Qiagen).

### Promoter reporter luciferase assay.

The 10T1/2 cells were seeded in a 96-well plate at a density of 5×10^4^ /ml, 150μl per well and incubated for 24 hours at 37°C with 5% CO2 to get 80–90% confluence. Transfection was performed using Lipofectamin with Plus Reagent (Invitrogen) based on the following plasmid ratio: the *Col1a2* promoter reporter (*Col1a2*_luc) or the *Col1a2* promoter reporter with CArG box mutation (*Col1a2*_*CArG_mut-luc*) 50 ng, internal control renilla luciferase reporter (*pRL_CMV*, Promega) 1ng, *pCGN_SRF* or *pCGN* mock 10 ng, *XFIF-Smad3* or *XFIF* mock 10 ng [24]. The luciferase activity assay was conducted 24 hours after transfection using the Dual-Luciferase Reporter Assay System (Promega, E1910) on a Veritas^™^ Microplate Luminometer (Promega). Each transfection was carried out in triplicates on the same plate and the analyses were based on experiments on three independent experiments.

### Chromatin immunoprecipitation assay (CHIP).

10T1/2 cells after pCGN-SRF transfection for 48 hours were prepared for CHIP assay using the EZ-ChIP kit (Millipore). The sonicated reaction mixtures were subject to pull-down by 1 μg of either a rabbit anti-SRF antibody (Santa Cruz, sc-335) or a control rabbit IgG antibody (Invitrogen) followed by the reversal of cross-link. The primer pair for both conventional PCR and real-time PCR was 5′-AGTGAAGCGGGACTGGACA and 5′-GGCTTTCGAGGGGGAACTC; the PCR product containing the CArG box was 201 bp.

### Electrophoresis mobility shift assay (EMSA).

Nuclear fraction was harvested from COS-7 cells using the NE-PER reagent (Pierce) 48 hours after transfection with pCGN-SRF expression plasmid. The 20-bp CArG box probe from the *Col1a2* promoter was 5′-TGCTTCCAAACTTGGCAAGG with 5′ IRDye labeling (originally synthesized by LI_COR, now by IDT); the CArG box mutant oligo sequence is 5′-TGCTTCCCAACTTGGCAAGG. The antibody used for supershift was rabbit anti-SRF (Santa Cruz, sc-335). The reaction mixtures of EMSA were prepared using the Odyssey Infrared EMSA Kit (LI_COR) according to the manufacturer’s protocol. The reaction mixtures were resolved on a 6% DNA retarding gel (Invitrogen) followed by visualization on an Odyssey Dual-Mode Imaging system (LO_COR).

### Statistics.

Five *Sm22*^−/−^ mice and five *Sm22*^*+/+*^ littermates were used in histology, immunohistochemistry and RT-PCR analyses. Values are means ± SE. Statistical analyses were performed using SPSS13.0 software. Student t-test was applied to evaluate differences in all experiments and differences were considered significant at p < 0.05.

## Results

### Carotid injury triggers the upregulation of COL1A2 expression in the medial layer of Sm22^−/−^ mice.

Previous studies from our lab illustrate that SM22 deficiency leads to arterial inflammation and chondrogenesis, accompanied by extracellular matrix (ECM) remodeling in response to carotid injury [15, 16, 18]. Given that fibrosis can stimulate both inflammation and chondrogenesis, we examined the expression of COL1A2, a fibrotic marker indicative of type I collagen, in *Sm22*^−/−^ mice in comparison to their *Sm22*^*+/+*^ littermates.

**Figure 1:**
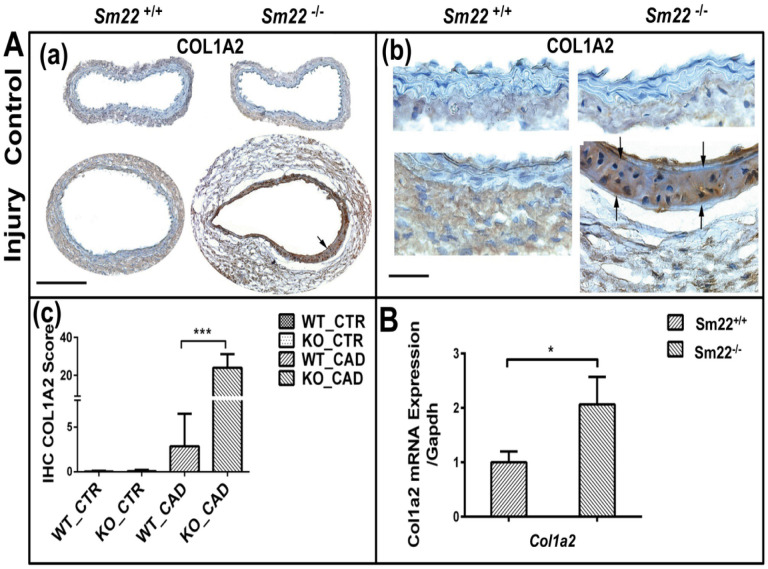
SM22 deficiency increases COL1A2 expression in Sm22−/− mice two weeks after carotid denudation. (A) Immunohistochemical (IHC) assays showing COL1A2 expression in carotid sections (CAD) of *Sm22*^−/−^(KO) and their littermates *Sm22*^+/+^ (WT) at 100X (a) and 400X (b). Bar: 100 μm (a) and 20 μm (b). (c) Quantification of positive signals at 100X magnification in the media of carotids from five *Sm22*^−/−^ and their littermates *Sm22*^+/+^ mice. Representative positive signals (brown) are indicated by arrows. (B) RT-PCR assay shows the relative mRNA level of *Col1a2* in the carotids of *Sm22*^−/−^ and their *Sm22*^+/+^ control mice two weeks after denudation. Values represent means ± SE from five pairs of mice. The asterisk (*) indicates p < 0.05.

Immunohistochemical (IHC) analysis uncovered a noteworthy increase in the expression of *COL1A2* in the smooth muscle cell (SMC) layer of the vessel wall, harvested two weeks post-carotid denudation (Fig. 1A). In line with this increased protein expression, *Col1a2* mRNA level was also elevated in the injured carotids of *Sm22*^−/−^ mice as opposed to their *Sm22*^*+/+*^ counterparts (Fig. 1B). These findings suggest that the transcriptional activation of *Col1a2* may contribute to the upregulation of *Col1a2* expression in *Sm22*^−/−^ carotids.

### Col1a2 is a putative SRF target gene and SRF binds and transactivates the CArG box in the Col1a2 promoter.

**Figure 2:**
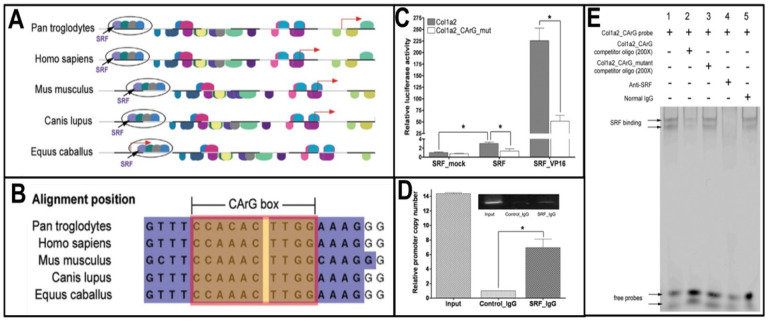
SRF transactivates the Col1a2promoter by binding to the CArG box. **(A)** An evolutionally conserved SRF binding site was identified by bioinformatics analyses of the sequence in the *Col1a2* promoter in several species. (**B**) The sequence alignment of the SRF binding site (the CArG box and its flanking sequence). (**C**) The luciferase assay shows that SRF or SRF-VP16 transactivates the luciferase reporter driven by the *Col1a2* promoter, not by the *Col1a2* promoter with mutation at the CArG box. (**D**) ChIP-qPCR assay using an SRF antibody detected a 201 bp *Col1a2* promoter sequence containing the CArG box by the qPCR. The inserted inset panel showed the amplified PCR fragments separated by gel electrophoresis. (**E**) Nuclear fraction from SRF transfected COS7 cells was used for EMSA using a 20 bp probe containing the CArG box in the *Col1a2* promoter. A non-labeled competitor oligo (lane 2), a non-labeled competitor oligo harboring a CArG box mutation (lane 3), the anti-SRF antibody (lane 4), and the IgG control (lane 5) was then added before EMSA. Values in (C) and (D) are means ± SE from three independent experiments. The asterisk, *, indicates p < 0.05.

To investigate the molecular mechanism that regulates the transcription of *Col1a2*, we performed bioinformatic analyses using tools including the MatInspector and the VISTA genome browser. We identified a transcription module that contains an evolutionally conserved CArG box binding site (Fig. 2A–2B). SRF is known to bind to the CArG box to regulate the transcription of genes involved in a variety of pathophysiological processes [22, 23, 27]. To confirm that SRF could activate the *Col1a2* promoter, we cloned the mouse *Col1a2* promoter into the pGL3Basic promoter luciferase reporter plasmid and generated a corresponding CArG box mutant. The luciferase reporter assay shows that SRF upregulated the *Col1a2* promoter activity by 3.5 fold over its mock control; and that this activation was reduced significantly in the *Col1a2* promoter with the CArG box mutant (Fig. 2C, middle 2 columns). Consistent with this result, SRF-VP16 drastically increased activities of the wild type the *Col1a2* promoter, not the *Col1a2* promoter with the CArG box mutant (Fig. 2C, right 2 columns). To determine whether SRF binds to the CArG box region of the *Col1a2* promoter *in vivo*, we performed ChIP assay. Both real-time PCR (Fig. 2D) and regular PCR (Fig. 2D, the inset panel) reveal that SRF antibody precipitated a significant amount of *Col1a2* promoter chromatin containing the putative CArG box. To further investigate whether SRF can bind directly to the putative CArG box, we performed the EMSA assay using a 20 bp oligo probe containing the CArG box in the *Col1a2* promoter. The binding of SRF to the probe was competitively inhibited by the excess amount of unlabeled oligo of the same sequence (Fig. 2E, lane 2) but not by the CArG mutant oligo (Fig. 2E, lane 3). In addition, the SRF-probe complex was disrupted by the SRF antibody (Fig. 2E, lane 4) but not by the IgG control (Fig. 2E, lane 5). Taken together, these results support that *Col1a2* is a direct target of SRF and can be activated by SRF binding to the CArG box in its promoter.

### SRF and SMAD3 cooperate to activate the Col1a2 promoter.

**Figure 3:**
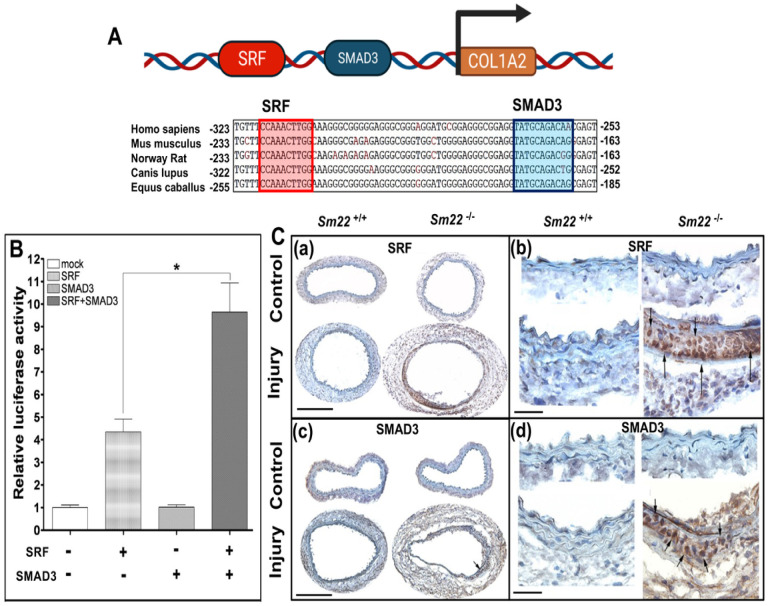
SRF and SMAD3 cooperate to activate Col1a2 promoter transcription. (A) Schematic illustration demonstrates the high conservation of SRF (the left side box) and SMAD3 (the right side box) binding sites at −163 and −233 upstream to the Transcription Starting Site (TSS) in the *Col1a2* promoter. Binding sites for SRF and SMAD3 were identified and compared across Homo sapiens, Mus musculus, Norway rat, Canis lupus, and Equus caballus using ChIP-seq Gene Transcription Regulation database (GTRD) and eukaryotic promoter database (JASPR). (B) Activation of the *Col1a2* promoter luciferase reporter by SRF (column 2) was significantly enhanced by co-transfection with Smad3 and SRF (column 4). Values represent means ± SE from three independent experiments. The asterisk (*) indicates p < 0.05. (C) Immunohistochemical (IHC) staining revealed increased expression of SRF (a, b) and SMAD3 (c, d) in the injured carotids of *Sm22*^−/−^ mice (lower row), not observed in their littermate control *Sm22*^+/+^ mice (upper row). The accumulation of SRF and SMAD3 in the nuclei of the smooth muscle layer of *Sm22*^−/−^ mice with injury (b and d, lower row at 400X) is indicated by arrows. (a, c) 100X, bars: 100 μm; (b, d) 400X, bars: 20 μm.

Our previous study shows that SRF interacts with Smad3 to regulate gene transcription [24]. To determine whether SRF and SMAD3 regulate the transcription of *Col1a2* in VSMCs, we performed bioinformatic analyses of the *Col1a2* promoter. Motif finding analysis of eukaryotic promoter database (EPD) identified evolutionarily conserved SRF and SMAD3 binding sites at − 163 and − 233 upstream to the TSS within the *Col1a2* promoter in the promoter region of mouse *Col1a2* gene (Fig. 3A). ChIP-seq analyses using the Gene Transcription Regulation Database (GTRD) detected the binding of both SRF and SMAD3 to the promoter regions of human *COL1A2* and mouse *Col1a2* in mouse aortic SMCs. We also observed their binding to the conserved human *COL1A2* gene in several human cancer cell lines and embryonic stem cells. The SRF and SMAD3 bind sites found in ChIP-seq databases overlaps with those SRF and SMAD3 binding sites (red and blue boxes) in the promoter region of mouse *Col1a2* gene using the Motif finding analysis tool for the eukaryotic promoter database (EPD).

Since SMAD3 binding site is near the CArG box in the promoter region of *Col1a2* gene, we were promoted to examine whether SMAD3 participates SRF–mediated transactivation of the mouse *Col1a2* promoter. We thus co-transfected SRF and SMAD3 with the luciferase reporter driven by the *Col1a2* promoter. Although SMAD3 alone did not significantly increase the promoter activities, combination of SRF and SMAD3 boosted *Col1a2* promoter activities about 10 folds while SRF only activates the *Col1a2* promoter activities to about 4.5 folds (Fig. 3B). These results demonstrate that SRF and SMAD3 can bind to *Col1a2* chromatin to exert synergetic effect on the transcriptional activation of the *Col1a2* promoter.

The association between the activation of SRF transcriptional activity, TGF-β signal pathway, and the nuclear translocation of SRF and SMAD3 is well-established. We conducted immunohistochemistry assays on *Sm22*^−/−^ mice injured arterial wall to examine the expression of SRF and SMAD3. Our findings reveal that both SRF and SMAD3 are minimally expressed in the vessel walls of both *Sm22*^−/−^ and its wildtype control mice without injury (Fig. 3C). However, two weeks post-injury, we observed a significant increase in the expression of both SRF and SMAD3 in the carotid media of *Sm22*^−/−^ mice (Fig. 3C). The nuclear presence of SRF and SMAD3 is indicated by arrows. Collectively, these findings suggest that injury activates the SRF and SMAD3-mediated canonical TGF-β signal pathway, promoting the transcription of *Col1a2* in the carotids of *Sm22*^−/−^ mice.

## Discussion

**Figure 4:**
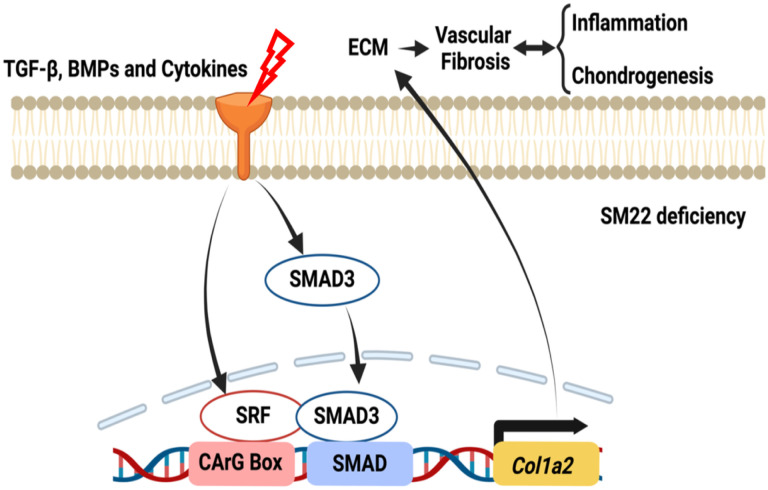
Schematic illustrating the role of SM22 deficiency in vascular fibrosis following arterial stress. Vascular injury triggers stress signals including TGF-β, BMP2, and proinflammatory cytokines, leading to the translocation of SRF and Smad3 into the nucleus to activate *Col1a2* transcription. The increased collagen in the ECM leads to vascular fibrosis, which engages in reciprocal interactions with vascular inflammation and chondrogenesis, contributing to vascular remodeling.

To demonstrate the active roles of downregulation of contractile proteins in arterial pathogenesis, we and a series of independent studies have demonstrated that loss of SM22 led to various interrelated pathogenic processes ranging from proliferation, migration, inflammation, chondrogenesis to senescence [14–19, 28]. Here we show that SM22 deficiency also induces vascular fibrosis via the activation of the transcription of *Col1a2* by SRF and SMAD3 in *Sm22*^−/−^ mice in response to vascular injury (Fig. 4).

This study stems from our pursuit to identify a common regulatory mechanism that links both pro-inflammatory and chondrogenic phenotypic changes in vascular smooth muscle cells (VSMCs) following injury in *Sm22*^−/−^ mice [15, 16]. We consider *COL1A2*, an extracellular matrix (ECM) protein, as a potential candidate. This choice is based on the upregulation of type I collagen expression in the pre-chondrogenic stage, as demonstrated in previous studies [20, 21], which contributes to chondrogenesis. Additionally, Type I collagen has the capability to induce a pro-inflammatory phenotype in VSMCs [29]. As anticipated, the immunohistochemistry (IHC) and reverse transcription-polymerase chain reaction (RT-PCR) results illustrated a substantial expression of *Col1a2* in injured *Sm22*^−/−^ carotids (Fig. 1). Given that increased Col1a2 is a marker of vascular fibrosis, its elevated expression strengthens the association of vascular fibrosis with the observed proinflammatory and chondrogenic phenotypic changes in VSMCs. This finding suggests that *Col1a2* may serve as a valid mediator connecting vascular fibrosis with inflammation and chondrogenesis during vascular remodeling in *Sm22*^−/−^ mice.

Since there was an elevation of *Col1a2* transcription in *Sm22*^−/−^ carotids after injury, we next sought to characterize the molecular mechanism to regulate the transcription of *Col1a2* in VSMCs (Fig. 2–3). The identification of a SRF binding site in the *Col1a2* promoter from routine bioinformatics analysis offered a clue of possible role of SRF in activating *Col1a2* transcription since SRF is shown to promote inflammation [23, 30]. *Col1a2* is a true SRF target gene, which is further confirmed by the promoter reporter, ChIP and EMSA assays (Fig. 2). In agreement with activation of *Col1a2* by SRF, IHC analysis revealed prominent SRF nuclear localization in injured *Sm22*^−/−^ carotid (Fig. 3C). This result is consistent with the binding of SRF to the *Col1a2* promoter reported in cardiac myofibroblasts in response to myocardium infarction [31].

Col1a2 is a known SMAD3 target in the TGF-β pathway [32–35]. SMAD3 also promotes SOX9 and CBP/p300 interaction in chondrogenesis [36]. Our previous study shows that SMAD3 interacts with SRF to activate *Sm22* transcription [24]. Since SMAD3 and SRF binding sites in the Col1a2 promoter are in close proximity [33], SRF may form a transcriptional module with SMAD3 to activate Col1a2. This hypothesis is supported by the synergistic effect of SRF and SMAD3 on *Col1a2* promoter activity (Fig. 3C, Fig. 4).

It is intriguing to observe that disrupting a contractile protein SM22 also demonstrates a novel role in regulating fibrosis in addition to its known impacts on VSMC inflammation and chondrogenesis[15, 16, 18]. These findings support the concept of VSMC plasticity in response to vascular stress, highlighting the central role of SRF in intricately regulating VSMC phenotypic changes through interactions with other key transcription factors such as Myocd, Elk1, NF-κB, Sox9, and Smad3 [18, 24, 37–40]. Given Smad3’s affinity for the SRF binding domain in Myocd [37, 41–43], further research should examine whether Smad3 competes with Myocd for binding to SRF. This potential competition could reveal mechanistic insights into TGF-β signaling responses during vascular fibrosis. The molecular mechanisms discovered in this study suggests that SM22 could be a potential therapeutic target for preventing fibrosis.

## Data Availability

All data generated during this study are included in this published article.

## Key Message

- SM22α disruption leads to vascular fibrosis upon arterial injury of *Sm22*^−/−^ mice.
- *Col1a2* transcription relies on the CArG-SRF-SMAD3 regulatory module in vascular cells.
- SM22α deficiency causes vascular remodeling including fibrosis, inflammation, and chondrogenesis.
- SM22α could be a target for preventing vascular fibrosis.
